# Draft Genome Sequences of Four Closely Linked Vibrio vulnificus Isolates from the Biotype 1 Environmental Genotype

**DOI:** 10.1128/genomeA.01317-14

**Published:** 2015-01-15

**Authors:** Kelsey E. Phillips, Matthew J. Schipma, Karla J. F. Satchell

**Affiliations:** a Department of Microbiology-Immunology, Northwestern University, Feinberg School of Medicine, Chicago, Illinois, USA; b Next-Generation Sequencing Core Facility, Northwestern University, Feinberg School of Medicine, Chicago, Illinois, USA

## Abstract

Biotype 1 of Vibrio vulnificus, which causes severe invasive intestinal and wound infections, is split into two genotypes with all previously sequenced clinical isolates from the C genotypes. We report here the whole-genome sequencing of two clinical isolates and two closely linked oyster isolates from the E genotype for comparative studies.

## GENOME ANNOUNCEMENT

Vibrio vulnificus is a seafood-associated pathogen found in coastal waters and can cause gastroenteritis, primary septicemia, and necrotizing fasciitis in food-borne and wound infections, respectively. This pathogen is often found in association with oysters and other shellfish and is thus of particular risk to both human handlers and consumers of raw seafood (1). The mortality rates for infection are high, with 50% and 15% for sepsis and wound infection, respectively (2), and infections are often difficult to treat. The characterization of the virulence factors of this pathogen is of particular importance, as the incidence of infection increases with increased water temperatures due to global warming (3–5). V. vulnificus is a highly diverse species (6) classified into three biotypes. Biotype 2 is predominantly associated with disease in eels, and biotype 3 is a recently emerged group of outbreak strains currently geographically limited to Israel (7, 8). Biotype 1 strains cause most human infections and are bifurcated into two separate but closely related phylogenetic genotypes based on the *vcg* gene. The environmental (E-type) strains are primarily isolated from harvested seafood or open waters, and the clinical (C-type) strains are isolated from human infections (9). Multilocus sequence typing (MLST) studies have a revealed a group of C-type strains that recently emerged from a cluster of E-type strains (10), leading us to hypothesize that these C-type strains have a genetic factor that is important for disease in humans that the E-type strains are lacking. Due to the vast difference in the gene contents between the C-type and E-type strains (2, 11), it is difficult to compare across such diverse strains. Therefore, we sequenced the genomes of two clinical isolates in the E genotype along with those of two oyster isolates that are closely linked by MLST.

In this paper, we describe the draft genome sequences of two clinical and two environmental V. vulnificus strains. ATL 6-1306 (CDC 9031-96) and ATL 71503 (CDC 9075-96) are strains isolated from separate patients in Florida in 1996. Strain 99-578 DP-B1 is an environmental strain isolated from an oyster in Louisiana in 1998, and 99-796 DP-E7 is an oyster isolate from Florida in 1998 (10). Genomic DNA was extracted according to the manufacturer’s instructions using the Qiagen DNeasy genomic DNA prep kit for Gram-negative bacterial cultures. DNA libraries were generated by the Northwestern University Genomics Core Facility and sequenced using the Life Technologies Ion Torrent PGM technology. The 318 Chip paired with a 400-bp library was chosen for maximum coverage, as V. vulnificus has a large genome (~5 Mbp). Newbler (GS *de novo* Assembler version 2.7) from 454 Life Sciences was used to assemble each of the genomes, and these were then aligned to the genome of V. vulnificus clinical isolate CMCP6 (Biosample SAMN02603130) using Torrent Mapper (TMAP) version 3.4.1. The average coverages for the genomes ranged from 53× to 65×, and these genomes were subsequently annotated using the Rapid Annotations using Subsystems Technology (RAST) SEED-based prokaryotic genome annotation service (12).

### Nucleotide sequence accession numbers.

The whole-genome shotgun projects have been deposited at DDBJ/EMBL/GenBank under the accession numbers JSVD00000000, JSWN00000000, JSWO00000000, and JSWP00000000. The versions described in this paper are JSVD01000000, JSWN01000000, JSWO01000000, and JSWP01000000.
